# Many Questions Remain Unanswered About the Role of Microbial Transmission in Epizootic Shell Disease in American Lobsters (*Homarus americanus*)

**DOI:** 10.3389/fmicb.2022.824950

**Published:** 2022-05-06

**Authors:** Suzanne L. Ishaq, Sarah M. Turner, M. Scarlett Tudor, Jean D. MacRae, Heather Hamlin, Joelle Kilchenmann, Grace Lee, Deborah Bouchard

**Affiliations:** ^1^School of Food and Agriculture, University of Maine, Orono, ME, United States; ^2^Aquaculture Research Institute, Orono, ME, United States; ^3^Cooperative Extension, University of Maine, Orono, ME, United States; ^4^Department of Civil and Environmental Engineering, University of Maine, Orono, ME, United States; ^5^School of Marine Sciences, University of Maine, Orono, ME, United States; ^6^Department of Neuroscience, Bowdoin College, Brunswick, ME, United States

**Keywords:** microbial community assembly, environmental microbiome, epizootic shell disease, shell associated community, Atlantic Ocean microbiome

## Abstract

Despite decades of research on lobster species’ biology, ecology, and microbiology, there are still unresolved questions about the microbial communities which associate in or on lobsters under healthy or diseased states, microbial acquisition, as well as microbial transmission between lobsters and between lobsters and their environment. There is an untapped opportunity for metagenomics, metatranscriptomics, and metabolomics to be added to the existing wealth of knowledge to more precisely track disease transmission, etiology, and host-microbe dynamics. Moreover, we need to gain this knowledge of wild lobster microbiomes before climate change alters environmental and host-microbial communities more than it likely already has, throwing a socioeconomically critical industry into disarray. As with so many animal species, the effects of climate change often manifest as changes in movement, and in this perspective piece, we consider the movement of the American lobster (*Homarus americanus*), Atlantic Ocean currents, and the microorganisms associated with either.

## Introduction

The American lobster, *Homarus americanus* (family Nephropidae), is an iconic and delicious (authors, personal communications) marine crustacean found along coastal waters in the Northwestern Atlantic Ocean, from latitude ~56° N, present day Labrador Peninsula in Canada, down to latitude ~34° N, present day North Carolina, in the United States. Lobsters have a hard carapace, which they molt regularly to grow, and large but unequally sized front claws used for feeding and defense (Aiken, 1980). While not all lobster species have been extensively studied, generally, lobsters are mid-trophic level community members, who transfer energy from primary producers further up the food chain (Radhakrishnan et al., 2019). In addition to exhibiting some grazing of algae and plants and predation on various bivalves and crustaceans, lobsters primarily scavenge detritus and turn over decaying material in the ecosystem (Radhakrishnan et al., 2019). In the past few decades, increasingly prevalent infectious disease, a slow northward migration, and stress from warming or acidifying waters have piqued research interests, and a significant amount of research on lobster biology and ecology has been accomplished.

Marine ecosystems are an untapped opportunity for metagenomics, metatranscriptomics, and metabolomics to increase our knowledge of ocean and lobster-associated microbiomes—the collective genomes of the bacteria, fungi, archaea, protozoa, and viruses in a community. For example, phages are known to infect and destroy large numbers of marine microorganisms in very short periods of time, effectively remodeling the microbial ecosystem (Breitbart et al., 2018). Further, horizontal gene transfer, whether mediated by phage infection or otherwise, is extremely common in hosts (Degnan, 2014) and marine systems (Nakamura, 2019). Metagenomics and whole-genome sequencing would allow for greater resolution of genomic data and the ability to better identify microbial individuals. Coupled with time-resolved sampling, this would allow for better tracking of individual microbiota over time and space. Changing environmental conditions are known to rapidly effect changes in microbial gene expression, and plasticity in biochemical substrates allows the same microbial individual to act very differently. Here, we consider the microbiological, biological, ecological, social, and economical impacts of the movement of lobsters, ocean currents, and the microorganisms associated with either.

### Slowly but Steadily, Lobsters Are Migrating Northward

Perhaps the most apparent change in movement is that of animal habitat. Lobsters prefer colder waters and become physiologically stressed after extended periods in waters warmer than 22°C. In the past several decades, densities have dramatically declined in coastal waters which have warmed, as lobster populations slowly migrate north at a rate of ~43 miles per decade (Pinsky et al., 2013). While the Gulf of Maine has seen a recent lobster population boom, attributed to this range shift, Gulf waters are also warming quickly and populations are expected to continue to march northward. Meanwhile, warming temperatures are altering animal health, lobster-associated microbial communities, and ocean microbial communities, further mechanisms for destabilizing the population.

American lobsters have long been part of the diet and livelihood of coastal communities, beginning with historical accounts through present day, including Beothuk, Eastern Abenaki, Mi’kmaq, Passamaquoddy, and Wampanoag peoples [The Centre for Indigenous Peoples’ Nutrition and Environment (CINE), 2017], as well as peoples in New England and Mid-Atlantic U.S., and the eastern maritime provinces of Canada. Currently, lobster catch, or landings, measures from ~10 million pounds per year in southern New England to ~140 million pounds per year from the Gulf of Maine, representing 79% of the total value caught by Maine fishermen (Atlantic States Marine Fisheries Commission, 2021; Department of Marine Resources, 2021) where the industry generates around half a billion dollars annually (Commercial Fishing Historical Landings Data, 2020). The lobster industry in Canada generates nearly 1 billion dollars annually (Government of Canada, Fisheries and Oceans Statistical Services, 2021). Intra-industry relations between indigenous and non-indigenous Canadian fishermen as well as negotiations between Maine fishermen and both marine mammal conservation groups and offshore energy producers have often turned contentious, illustrating how vital the lobster industry is along the eastern seaboard. The lucrative nature of the current market, combined with high barriers to entry, ongoing territorial disputes, and conflicts with state and federal policy makers have led to prolonged regulatory and social battles in many coastal areas (Levinson-King, 2020). In recent decades, a myriad of reasons, including overfishing of other lucrative species and climate change driven population shifts, have led to the siloing of Maine’s lobster fishers, with many families and communities depending primarily on lobster for income (Steneck et al., 2011; Stoll et al., 2016). In Southern New England, where lobster abundance declined by around 70% in recent years, state managers suggested a complete closure of the fishery (Steneck et al., 2011). In Maine, where rural communities tie their cultural identity to the lobster fishery and have little flexibility to pursue other opportunities (Stoll et al., 2016), a similar collapse of the industry would have significant and widespread social and economic impacts, in addition to the ecological impacts.

### Epizootic Shell Disease Is More Prevalent Over Time and With Increased Temperature

Considering emerging diseases, epizootic shell disease (ESD) has been particularly perplexing since it was first observed in *H. americanus* in the mid-1990s and has dramatically increased in prevalence along U.S. coastal waters (Castro et al., 2012). Shell diseases in crustaceans are common, generally cause softening or pitting of the shell, and depending on the disease, have microbial or environmental causes (Tlusty et al., 2007; Sweet and Bateman, 2015) In ESD, shell degradation can lead to lesions with poly-microbial communities present but it does not spread to internal tissues (Watson, 2005). Research studies have linked ESD to disease-associated mortality (Hoenig et al., 2017; Groner et al., 2018), but it remains to be determined if ESD microbial communities are the direct cause of mortality or if shell degradation increases the susceptibility of the animal to other infections, predation, and/or physical damage. Chitinolytic bacteria were originally suspected of degrading shells, and though present and active on shells, microbial chitinase enzymes are not as active on shells as microbial proteolytic and cellulolytic enzymes (Bell et al., 2012). Disease diagnosis is difficult prior to the development of physical signs of disease, and many potential bacterial pathogens have been identified ubiquitously on apparently healthy lobsters, (e.g., Meres, 2016; Bouchard, 2018; Ishaq et al., 2021a) or in marine environments (Watson, 2005). A study used amplicon sequencing and did not find putative ESD pathogens to also exist in tank biofilms and that putative pathogens may be associated with more disease symptoms but not disease onset (Whitten et al., 2014). Rather than being associated with a different community, ESD is associated with having more bacterial growth present, as confirmed by scanning electron microscopy of shells (Tlusty et al., 2007).

In decades of research, the causative microbial or viral agent for ESD has not been identified, though many studies attempted to Castro et al. (2012). Previous research has used several regions of the 16S rRNA gene to identify bacterial species using denaturing gradient gels (e.g., Chistoserdov et al., 2012; Quinn et al., 2013) or communities using amplicon sequencing (e.g., Whitten et al., 2014; Meres, 2016; Reardon et al., 2018), and one study used amplicon sequencing on the 18S rRNA gene to identify eukaryotes associated with ESD (Quinn et al., 2009). One study using denaturing gradient gels also used infection models under experimental conditions to determine if putative infectious bacteria might be causative agents (Quinn et al., 2012). To our knowledge, no study has explored the microbial community using shotgun sequencing metagenomics techniques to identify microbial genomes of any bacteria, fungi, archaea, protozoan, or virus present or using shotgun sequencing metatranscriptomics to identify active gene transcriptions from microorganisms present, from healthy and diseased shells to investigate those functional changes.

Further, lobsters do not respond to ESD as they respond to typical bacterial infections, although there are some gene expression changes in ESD lobsters which indicate less growth and more innate immune activity (Tarrant et al., 2010). *In vitro* antimicrobial activity of hemolymph collected from lobsters was not higher in ESD lobsters compared to healthy controls (Bouchard, 2018) and was lower in both ESD and healthy lobster hemolymph compared to hemolymph spiked with the bacteria *Escherichia coli* as a positive control (Bouchard, 2018). Assuming that the lack of response is because the ESD-associated microbial community is recognized by the lobster as commensal, this lends weight to the theory that shell microbial community taxonomic structure is not changed, only microbial function is. The immune response dynamic was suppressed when hemolymph was collected from ESD lobsters that had been housed in warmer waters; *in vitro* antimicrobial activity was lowest in those kept in the warmest waters compared to medium or low temperatures (Dove et al., 2005; Bouchard, 2018). ESD progression is faster in lobsters housed under warmer temperatures for prolonged periods (Barris et al., 2018) and, regardless of when or where the study took place, ESD is more prevalent in ocean waters which are routinely above 22°C (Glenn and Pugh, 2006; Tanaka et al., 2017; Groner et al., 2018). Studies that use higher resolution of spatial patterns of ESD prevalence and environmental conditions support the importance of water temperature, as ESD can be found even in areas of otherwise optimal lobster habitat (Tanaka et al., 2017).

## Interactions of Movement, Molting Behavior, and Temperature

Disease transmission dynamics of ESD have not been elucidated, and while the general consensus is that ESD is not transmitted from one animal to the next under laboratory conditions, this appears to be based on one note in a conference proceeding (Duboise and Moulton, 2005). This consensus is further tempered as most aquaculture-based lobsters are housed individually to prevent antagonism or cannibalism, which would preclude horizontal transmission of bacteria *via* direct contact, as well as *via* water if there is only one lobster per tank. Multiple lobsters may be housed per tank and separated by divider screens which allow water flow, which would theoretically spread the infection if it were simply a factor of localized exposure to certain microorganisms. There is also a general consensus that it is too difficult for tank systems to replicate the complex factors which would contribute to disease transmission in the wild. Further, because tank systems regularly filter water, possible causative agents could be removed from the tank system over time. In a study of wild-caught lobsters which were group housed but individually separated by mesh screens which allowed water flow, ESD signs did not worsen over time in most lobsters nor did they appear on apparently healthy lobsters after being co-housed with ESD-affected lobsters for over a year (Bouchard, 2018).

In wild populations along the United States coast, daily lobster movement has not been documented to be a factor in spread of disease (Watson, 2005), as even very mobile lobsters tend to have localized ranges (Watson, 2005; Scopel et al., 2009), and migration from deeper, cooler water to shallower, warmer water, and back was not associated with the spread of ESD (Watson, 2005). Those lobsters which migrate long distances appear to move further south and tend toward shallower waters on the move (Watson, 2005), contrary to the hypothesis that diseased lobsters would seek cooler waters in which to recuperate. However, movement of lobsters and disease transmission may be more of a problem in other locations: lobsters along the Canadian coast have been documented to move farther (Campbell, 1986; Morse et al., 2018), and American lobsters were released in the late 1990s and became invasive around Scandinavia and Britain where they pose various risks to European lobsters, *Homarus gammarus* (Whitten et al., 2014).

Lobsters molt regularly to shed their outer carapace and grow a new, larger shell. Molting frequency is somewhat seasonal based on food availability and ocean temperatures, and typically takes place during summer (June to August) and again in fall (September to October). The molt itself leaves lobsters vulnerable to environmental conditions and may result in subsequent disease signs (Aiken, 1980; Howell et al., 2005; Groner et al., 2018). If conditions are not favorable, or if a female is egg-bearing, the time between moltings will be prolonged. Taking longer to molt can increase ESD progression (Groner et al., 2018). Warmer temperature can induce molting earlier in spring (Groner et al., 2018), and molting out of sync with typical seasonal conditions can increase the risk of ESD if summer temperatures were hotter or prolonged (Groner et al., 2018). In wild and cultured lobsters, molting can reduce or remove signs of ESD entirely; however, if the outer carapace and deeper layers have been damaged, the new shell may show scarring or deformities (Stevens, 2009), which if severe enough can prevent detachment of the old carapace. It is often in the process of molt that lobsters die from ESD-related issues.

There is another hypothetical mechanism for horizontal transmission which previous research has considered. Lobsters can be cannibalistic or in the process of molt, lobsters may consume shed carapaces (Aiken, 1980), including the associated microbial community, although uneaten molted shells are removed from aquaculture systems to prevent fouling tank water. Consuming a molted shell that contains potentially pathogenic microorganisms raises several questions. If ESD were caused by infectious or toxin-producing microorganisms, would consuming a carapace and associated microbial community be linked to morbidity and mortality in lobsters, as those hypothetical pathogens wreaked havoc internally? Various studies refute this possible mechanism, discussed in Tlusty et al. (2007). There is evidence that healthy Caribbean spiny lobsters (*Panulirus argus*) will avoid other spiny lobsters that are infected with a virus (Behringer et al., 2006), although *Homarus americanus* females do not avoid ESD-positive males in laboratory settings based on olfactory cues in the water (Rycroft et al., 2012).

## Warmer Water Could Alter How and Which Microorganisms Are Carried by Ocean Currents

Ocean microbial communities are diverse and dynamic, and coastal anthropogenic activities can alter the microorganisms present, nutrient cycling, and other ecological processes (Stewart et al., 2008; Nogales et al., 2011), as well as induce antimicrobial resistance (Chen et al., 2019). Ocean currents are known to curate marine microbial communities by geographic location (Cavicchioli, 2015) and depth (Zinger et al., 2011). Ocean currents were also implicated as driving the bacterial community assembled on the shell of topshell sea snails, *Phorcus sauciatus*, found in coastal Northeastern Atlantic Ocean waters around Europe and the Mediterranean (Sousa et al., 2021). Not only are currents transporting microbial communities, but the varying environmental conditions along the way may alter the microorganisms, including making them tolerant to higher temperatures (Doblin and van Sebille, 2016).

Air currents, too, transport bacterial and fungal communities which are assembled from land, water, or urban environments where the clouds were formed (Amato et al., 2017). Combined with local weather, aerosolized microorganisms contribute to disease outbreaks when environmental conditions cause animal and human hosts to be more susceptible to infection or to microbial transmission (e.g., coughing more during dust storms; Griffin, 2007). It is feasible that microbial transmission along air or water currents could contribute to removing, adding, or circulating disruptive microorganisms in coastal waters which affects lobsters. For example, land-sourced microbial communities which are transported in air (Mayol et al., 2017) or water runoff (Adyasari et al., 2020) change the microbial communities found in coastal ocean waters, and this could have a protracted effect in summer when water circulates more slowly. During the summer, the Gulf Stream slows along eastern North America as warming land temperatures alter winds and push them *across* coastal water currents on an east–west axis, rather than *with* water currents on a northward path (Roarty et al., 2020). Around Cape Cod, summer winds contribute to a recirculating eddy in Massachusetts Bay which does not mix with deeper water nearly as much as it does in winter (Robinson, 2005).

While the hypothesis of currents and ESD transmission was discussed in a regional conference panel (Robinson, 2005), and air and water currents have been extensively studied in coastal waters in this region, including for lobster larval dispersal, this has never been evaluated for currents and ESD *via* water or vectored by larvae. If climate change and/or disease affects lobster populations in warmer waters, will microbial dispersal along ocean currents bring disease from southern to northern lobster populations even before temperatures rise there? Ocean currents have been implicated in the rapid and far-reaching spread of infectious disease in marine animals (McCallum et al., 2003), including in corals (Dobbelaere et al., 2020), starfish (Aalto et al., 2020), and fish (Stene et al., 2014; Alaliyat et al., 2019), using agent-based, environmental-based, and combination modeling of empirical infection data. While not yet implicated in lobster shell diseases, there is a plausible scenario in which ocean currents flowing from southern New England, where disease is more prevalent (Glenn and Pugh, 2006; Tanaka et al., 2017) and warmer temperatures select hardier microorganisms (Mayers et al., 2016), bring taxonomically similar but functionally different microorganisms further north. Or, phage dispersal along currents could induce horizontal gene transfer and a change of activity in existing host-associated microorganisms (Degnan, 2014). Even if those microbial or viral travelers are not infectious, *per se*, those microorganisms would still be in a geographic position to take advantage of altered lobster homeostasis due to warming waters or thinner shells caused by increasing acidity.

Warming and acidifying ocean waters work independently, and conjunctively, to weaken lobster immune and physical defenses (e.g., shell quality) and make them more susceptible to infectious disease (Harrington et al., 2020). While host susceptibility does play a small role in ESD, published studies suggest it has more to do with structural capacity of the shell than a weakened immune response or other symptoms associated with heat stress in terrestrial animals, e.g., “leaky gut” (Tlusty et al., 2007).

Separate from environmental conditions and host susceptibility is the capacity for microbes to colonize hosts and transfer between them, which could temper disease transmission. In an elegantly simple microbial transmission model, a bacterium was inoculated into germ-free zebrafish (*Danio rerio*) to track immigration and emigration through an animal population. *Aeromonas veronii* was isolated from the gastrointestinal tract of conventional zebrafish with typical microbiota, and colony lines created in which the same bacteria were added to a new germ-free fish colony, re-isolated, and added to new fish for a total of 22 passages. As compared to the ancestral bacterial lines, these replicate lines were more “fish-associated” and were able to colonize more fish in a given period of time, as well as cultivate larger cell counts in those fish they colonized (Robinson et al., 2018). Robinson et al. made use of a modified *A. veronii* with increased capacity for genetic mutation, to better study adaptation to a host. However, given enough time and the right environmental conditions, it is likely that other microorganisms could become better at colonizing animals in aquaculture systems.

In the case of ESD, in which it appears that the same bacteria are present but forming larger and more complex biofilms, the assembly, and succession of that shell biofilm community over time is likely to affect transmission dynamics. Further, water temperatures affect biofilm formation and dynamics, and so do pollution and nutrients in water runoff that finds its way into coastal waters—even if the pollution itself is not affecting lobsters directly. For example, *Aquimarina* and *Thalassobius* species of bacteria have long been posited to be involved with ESD lesions (Chistoserdov et al., 2012). Both were found to be part of biofilms associated with plastic waste after incubation in sea water for 40 days, though both were rare until after a further 94-day incubation (Jacquin et al., 2021), implying they might join biofilms after initial colonizations.

## Marine and Aquaculture Environments Foster Different Dynamics Affecting Microbial Transmission

Marine and aquaculture environments offer contrasts in their dynamics which likely generate distinct mechanisms of selection for host disease resistance or susceptibility, for microbial transmission, and for pathogen virulence or symbiosis. Marine environments have wildly diverse microbial communities which turn over quickly and dramatically with phage infection (Breitbart et al., 2018), weather patterns (Angly et al., 2016), or human settlement pollution (Nogales et al., 2011; Chen et al., 2019). Marine environments are likely to select for traits which favor microbial transmission between different animal hosts, as well as for long-term survival in the water when moving between hosts (Rebollar et al., 2016). Host-associated and free-living *Aquimarina* species were not demonstrated to have different biochemical capacities, although species varied widely in their abilities to be commensal or pathogenic (Silva et al., 2019). Silva et al. further noted that while many host-associated microbial strains have reduced genome sizes compared to their free-living, wild-type counterparts, *Aquimarina* did not differ in genome size by isolation source and that it had a larger genome than most known marine isolates. This could imply that *Aquimarina* are particularly adept at switching from host- to environmental-associated as needed.

Aquaculture facilities may select for host-species specificity in enclosed production systems, in which the only hosts available are from a single species and most likely at the same lifestage. Aquaculture production, like any selective practice, has the potential to increase or decrease microbial virulence, discussed in Kennedy et al. (2016). Aquaculture systems can be static and refreshed, flow through with no recirculation (one-time pass through), or recirculating with mechanical and biofiltration. All aquaculture facilities present surfaces on which microbial biofilms might accumulate (de Carvalho, 2018). Over time, even with regular draining and cleaning, microbial inhabitants may increase their ability to attach to surfaces, produce exopolysaccharides and other film materials, and remain in the system. Even if these biofilm-formers are not infectious, an abundance of microbial biofilms can still generate toxins or other compounds which affect host health. For example, microbial biofilms can alter the chemistry of surfaces and prevent shellfish larvae from settling and continuing their development (Qian et al., 2007). While lobster larvae do not attach upon settlement, biofilms on surfaces can affect the hospitality of their local environment. Biofilms also generate planktonic microbial cells which may be ingested or come into contact with animals. Poly-microbial biofilms can offer long-term protection and safe harbor for infectious microorganisms, which may then be released into the tank system to cause sporadic outbreaks (Levipan et al., 2020). However, previous studies which track bacterial communities in tanks and lobsters have not demonstrated much correlation between bacterial concentration and health in spiny lobster phyllosoma (larvae; Bourne et al., 2004), or American lobster adults (Bouchard, 2018).

## Discussion: Where Can Aquaculture Draw Lessons From?

Aquaculture systems can both simplify and complicate marine host microbiomes, as they contain a fraction of the biological diversity—not to mention typically offering a single and nearly static ecosystem—which dramatically reduces microbial diversity in water and animals. For example, preliminary data indicate that bacterial communities on lobster shells have dramatically more bacterial diversity when coming from ocean waters versus having spent several months in filtered-and -recirculated-water tank systems, even with light exposure and water temperatures which mimic seasonal variation (Ishaq et al., 2021a,b). This effect can be beneficial for isolating and removing infectious disease from a population, especially if the causative agent is an obligate host-associated microorganism or virus.

However, this does not remove the possibility of infectious built-environment-associated microorganisms. Aquaculture systems are potentially open to similar problems faced by water management systems in human settlements: pipes and storage tanks can foster microbial communities (Borella et al., 2004; Douterelo et al., 2016; Ling et al., 2018), and surfaces could act as attachment points for biofilms—especially in pipes with valves or other connection points. No sterilization mechanism is completely effective forever, either, as evidenced by the selection of chlorine-tolerant microorganisms in disinfection systems for human settlement water treatment (Liu et al., 2018).

Nor does a depauperate tank microbial community allow animal hosts to accumulate a diverse host-associated community which may protect them from infections. For example, laboratory mice in the same cage will transfer microorganisms through physical contact and grooming behaviors; transfer *via* air, cage surfaces, or other objects (i.e., fomites); and through feces as mice are coprophagic. If small groups of animals are left in the same cage under clean laboratory conditions, eventually their microbial communities will homogenize between individuals in each cage (Hildebrand et al., 2013). Over time, the cages act like islands, and without an influx of new microorganisms, the collective microbial community in the different cages will experience drift or random changes to the abundance of those remaining microorganisms (Nemergut et al., 2013). Yet, microbial transmission within a tank system does not appear to be spreading ESD among tank mates, even when lobsters showing ESD signs were grouped together which would theoretically increase negative microbial exposures (Bouchard, 2018).

Probiotics have been considered as a treatment or preventative for ESD (Underwood, 2018), as many aquatic microorganisms and viruses are antagonistic toward other species. Probiotics might be effective as topical treatments for tank or shell bacteria; however, while a topical probiotic could hypothetically be effective, it would not be practical at a scale needed to make an appreciable difference in wild populations. Not only are aquaculture facilities lacking to house the number of diseased lobsters which are caught annually, but application as a topical treatment to diseased lobsters which are captured and released would be extremely labor intensive and creates the potential for ecological ramifications of the probiotic in the wild. Further, probiotics may not be effective if ESD is mediated by altered microbial activity of the typical shell microbiota. In that hypothetical scenario, then, a probiotic or even bacteriophage-based topical treatment would need to disrupt or remove the biofilm on shells to give lobsters time to heal and perhaps molt before reassembly. If molting times were predicted by geographic region and seasonal temperature fluctuations, this strategy could be implemented to time it with molting. Given the sheer number of lobsters which are captured each year, this would be a sizable and costly undertaking, but priority could be given to ovigerous females to maximize impact. Probiotics could also be implemented fairly easily in aquaculture systems, or in other short-term lobster holding tanks, though it may take weeks or months to see any improvement using microbial treatment alone. Tank environmental conditions could be manipulated subsequently to induce molting after receiving anti-biofilm treatments, but this alters the molting pace by a scale of weeks, not days.

Despite economically driven interest and decades of research on lobster biology, ecology, and microbiology, there are still unresolved questions about lobster microbial communities in general, and regarding ESD in particular. There is an untapped opportunity for metagenomics, transcriptomics, and metabolomics to be added to the existing wealth of knowledge, to more precisely track disease transmission, etiology, and host-microbe dynamics.

## Author Contributions

SI conceptualized and wrote this perspective. SI, ST, MT, JM, HH, JK, GL, and DB contributed to refining the scope and ideas, writing, and reviewing. All authors contributed to the article and approved the submitted version.

## Funding

This project was supported by the USDA National Institute of Food and Agriculture (Hatch project number ME0-22102, Ishaq) through the Maine Agricultural and Forest Experiment Station (publication number 3865).

## Conflict of Interest

The authors declare that the research was conducted in the absence of any commercial or financial relationships that could be construed as a potential conflict of interest.

## Publisher’s Note

All claims expressed in this article are solely those of the authors and do not necessarily represent those of their affiliated organizations, or those of the publisher, the editors and the reviewers. Any product that may be evaluated in this article, or claim that may be made by its manufacturer, is not guaranteed or endorsed by the publisher.
